# The complete chloroplast genome sequence of *Casearia glomerata* Roxb. (Malpighiales; Salicaceae) from Fujian, China

**DOI:** 10.1080/23802359.2021.1936235

**Published:** 2021-06-14

**Authors:** Hui Huang, Yanqiu Xie, Site Luo, Linting Zhang, Chuanyuan Deng, Zujian Chen

**Affiliations:** a College of Landscape Architecture, Fujian Agriculture and Forestry University, Fujian, PR China; b Faculty of Architecture and Engineering, Fujian College of Water Conservancy and Electric Power, Fujian, PR China; c School of Life Sciences, Xiamen University, Xiamen, PR China; d Island Research Center, Ministry of Natural Resources, Fujian, PR China

**Keywords:** *Casearia glomerata*, chloroplast genome, phylogeny

## Abstract

*Casearia glomerata* Roxb. is classified in Salicaceae and has a high economic value. Herein, we report the complete chloroplast (cp) genome of *C*. *glomerata* using Illumina sequence data. The cp genome is 156,809 bp in length and contains a large single-copy (LSC) region of 84,888 bp and a small single-copy (SSC) region of 17,039 bp separated by two inverted repeat (IR) regions of 27,441 bp each. It contained a total of 123 genes, with an overall GC content of 36.81%. The phylogenetic analysis showed that *C. glomerata* is closely related to *Casearia velutina*. This study provides important sequence information for species identification and its phylogenetic relationship in the Salicaceae

*Casearia glomerata* Roxb. is a shrub classified in the plant family Salicaceae. It is widely distributed from Central Himalayas to Fujian, Guangdong, Guangxi, Hainan, Taiwan, Yunnan in China (Yang and Zmarzty 2007). *Casearia glomerata* has yellow-green flowers and orange-red fruits, which possess a high economic value to garden cultivation (Chen 1953). Chloroplasts (cps) are essential in plant cells and play a crucial role in sustaining life (Xiong et al. 2009). Due to the high conservation of cp genomes compared to the nuclear and mitochondrial genomes, cp genome sequences have often been used for phylogenetic studies and species identification (Khan et al. 2010). Currently, no study had been published on the complete cp genome sequence of *C. glomerata*. Here, we performed high-throughput sequencing on a specimen of *C. glomerata* from China to determine its cp genome structure and evolutionary relationship to Salicaceae.

The fresh leaves of *C. glomerata* were collected from dense forests on the coast of Haitan Island in Fuzhou, Fujian Province, China (25°49′68″N, 119°71′18″E). The voucher specimen is deposited at Fujian Agriculture and Forestry University (No. FZ-FJ2020-10A, FAFU, HuiHUANG: HuiHUANG@fafu.edu.cn). The genomic DNA was extracted using Plant Genomic DNA Kit, DP305 (TIANGEN, Beijing, China). The sequencing library was produced using the Illumina Truseq^™^ DNA Sample Preparation Kit (Illumina, San Diego, CA) according to the manufacturer's recommendations. The prepared library was loaded on the Illumina Novaseq 6000 platform for PE 2 × 150 bp sequencing at Novogene (Beijing, China). The raw data were used to assemble the complete cp genome using the GetOrganelle pipeline (Jin et al. 2020). Genome annotation was performed with PGA (Qu et al. 2019) by comparing the sequences with the cp genome of *Casearia velutina*, GenBank Accession Number MN078141. The annotated genome sequence was deposited in GenBank under Accession Number MW428079.

The circular cp genome of *C. glomerata* was 156,809 bp and contains a larger single-copy (LSC) region of 84,888 bp in length, a smaller single-copy (SSC) region of 17,039 bp in length, and a pair of inverted repeats (IRs) of 27,441 bp. There were 131 genes predicted in this genome, of which 79 are protein-coding genes, 36 *tRNA*, and eight ribosomal *RNA* genes. The base content of the *C. glomerata* cp genome is A (31.21%), T (31.98%), C (18.70%), G (18.11%), and the overall GC content of the cp genome is 36.81%.

The cp genome of *C. glomerata* was aligned with other nine cp genomes of Salicaceae and *Arabidopsis thaliana* was designated as the outgroup to construct the phylogenetic tree. All complete cp genomes were aligned with the MAFFT version 7.388 using default settings (Katoh and Standley 2013). The phylogenetic analysis was conducted based on maximum likelihood (ML) analyses implemented in IQ-TREE version 2.1.2 with the TVM + F+R5 nucleotide substitution model, which was selected by ModelFinder (Kalyaanamoorthy et al. 2017; Minh et al. 2020). The support for the inferred ML tree was inferred by bootstrapping with 1000 replicates. The phylogenetic analysis suggested that *C. glomerata* is closely related to *C. velutina* (Figure 1). This cp genome will provide an important resource for addressing taxonomic issues and studying the molecular evolution of *Casearia.*

**Figure 1.**
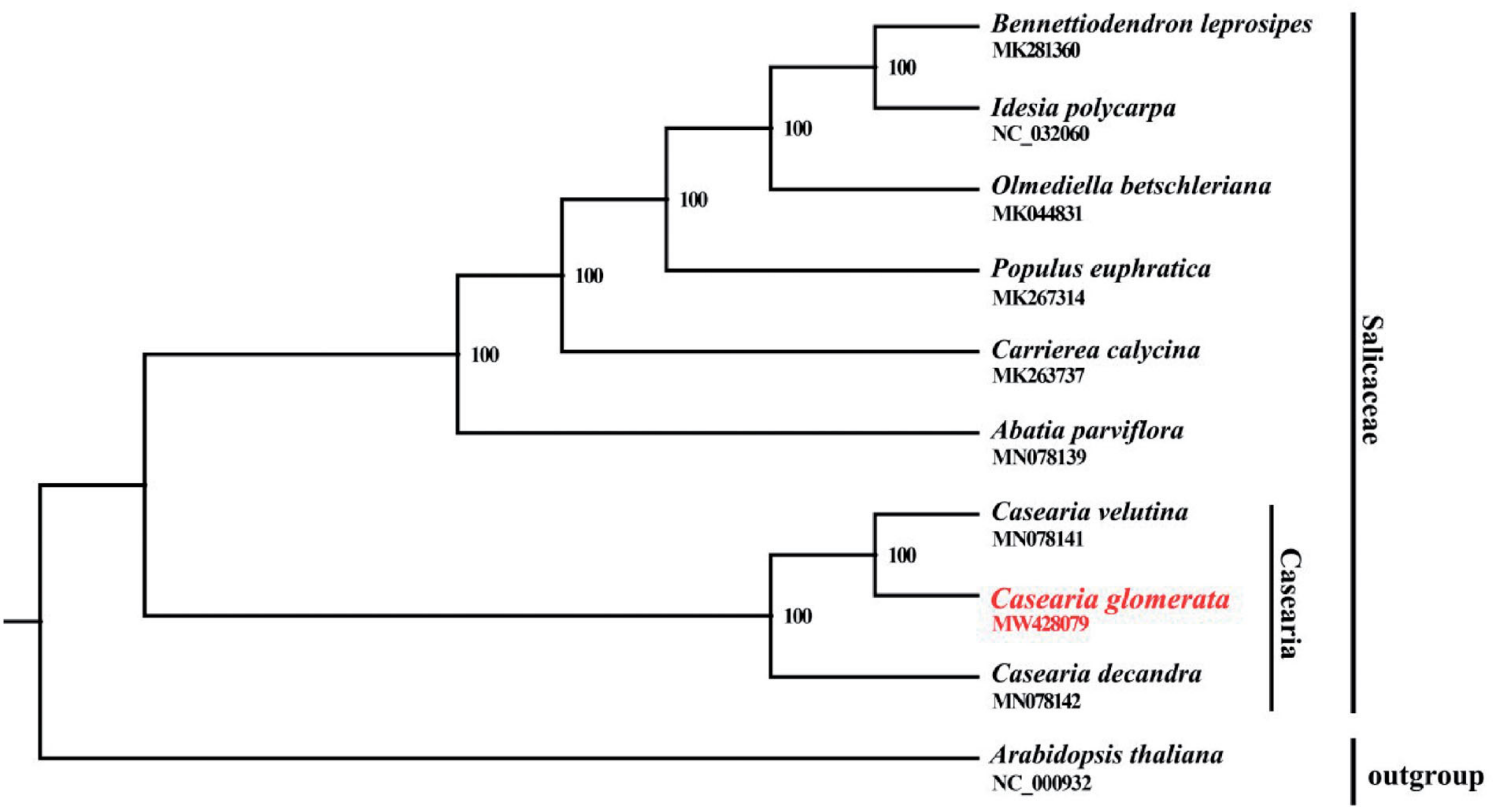
Maximum-likelihood (ML) tree based on nine cp genome sequences from the Salicaceae with *Arabidopsis thaliana* designated as outgroups. Numbers on the nodes are bootstrap values based on 1000 replicates. *Casearia glomerata* was marked in red.

## Data Availability

The genome sequence data that support the findings of this study are openly available in GenBank of NCBI at (https://www.ncbi.nlm.nih.gov/) under the accession no MW428079. The associated BioProject, SRA, and Bio-Sample numbers are PRJNA688994 SAMN17193100, and SRR13340381, respectively.
